# Practical tips for organizing challenge-based learning in biomedical education

**DOI:** 10.12688/mep.19755.1

**Published:** 2023-11-08

**Authors:** Farah R. W. Kools, Heleen van Ravenswaaij

**Affiliations:** 1 Center of Education and Training, Department of Biomedical Sciences, University Medical Center Utrecht, Utrecht University, Utrecht, Utrecht, 3508 GA, The Netherlands

**Keywords:** challenge-based learning, biomedical education, course organization

## Abstract

Challenge-based learning (CBL) in biomedical education can prepare health professionals to handle complex challenges in their work environments through the development and practice of problem-solving skills. This paper provides twelve practical tips for biomedical educators to implement CBL in their education. The intricacies of CBL are explained together with organizational tips, and multiple levels of student support to help students achieve CBL learning goals. Our aim is to promote CBL in biomedical education and to help students acquire valuable skills for post-graduation while working towards solving real societal needs.

## Introduction

Health professionals face complex problems and challenges in their work environments, for which adequate preparation is required during their biomedical education ( Mylopoulos *et al.*, 2016). To provide this preparation, challenge-based learning (CBL) can be implemented in biomedical curricula, giving students opportunities to practice and reflect on solving real-world problems ( Ward *et al.*, 2018). CBL is relatively new and is defined by Gallagher and Savage (2020) as a flexible approach that *“frames learning with challenges using multidisciplinary actors, technology enhanced learning, multi-stakeholder collaboration and an authentic, real-world focus.*” Although CBL resembles problem-based learning (PBL; Wood & Petocz, n.d.), there are important differences: 1) PBL starts with a given problem, whereas in CBL students formulate the exact problem, 2) PBL uses a product context and customer perspective, whereas CBL uses a transdisciplinary approach within a social context driven by value, and 3) PBL emphasizes team development, whereas CBL focuses on both team and individual development ( Kohn Rådberg, *et al.*, 2020).

In their international literature review, Gallagher and Savage (2020) provide a summary of the commonly agreed upon characteristics, challenges, and benefits of CBL. Benefits for students include the opportunities to network, apply skills in a real-world environment, practice multidisciplinary teamwork, tolerate ambiguity and uncertainty, and improve their problem-solving and technical skills, as well as deepening their knowledge. Additionally, working partnerships between academia and university are mentioned. However, in their review the authors also highlight the difficulties of reaching a consensus on a single CBL approach ( Gallagher & Savage, 2020).

This paper aims to offer practical suggestions for organizing CBL in biomedical education, drawing from our five years of experience with developing, executing, and evaluating CBL at the Graduate School of Life Sciences of Utrecht University in the Netherlands. Given the possible variations of CBL approaches, we describe different implementation choices that could be considered and adapted to local contexts and learning goals. These suggestions are intended to assist other biomedical educators in effectively incorporating CBL into their educational settings.

## Twelve tips

***1. Choose a CBL framework***

A framework provides structure for the design and execution of all CBL activities. When designing our CBL activities, we used the “ *Apple Classrooms of Tomorrow*” framework from Nichols, Cator, & Torres (2016). In this framework there are three distinct phases: 1) engage; students identify a complex real-world problem and define an actionable challenge, 2) investigate; students research their challenge to gain in-depth understanding, and 3) act; solutions are designed and tested. Additionally, we integrated design thinking into this CBL framework, which has previously been used in healthcare challenges (see the twelve tips by Wolcott *et al.*, 2021). Design thinking consists of a series of steps: 1) invest time to understand the problem extensively (divergent thinking), 2) define a specific challenge within the problem (convergent thinking), 3) explore possible solutions (divergent thinking), and 4) prototype and test the chosen solution (convergent thinking), otherwise known as the double diamond model.

We supported students during both divergent and convergent steps by providing them with tools and exercises. Divergent thinking requires being open and creative. Brainstorming techniques such as the “ *Six Thinking Hats*” or “ *Wishful Thinking*” can be used to encourage this. Convergent thinking involves narrowing down options and making logical decisions. Strategies such as combining similar ideas and voting for the best one or using an impact-effort matrix can aid in this process. During both divergent and convergent phases, we found that stimulating students to include stakeholder perspectives was highly motivating and ensured that creative directions remained on-topic and feasible.

***2. Select a complex real-world problem***

A real-world problem serves as the starting point for CBL and is in our experience the reason many of our students choose to participate. Gallagher and Savage (2020) identified three CBL characteristics in relation to this: 1) choose a global theme, 2) define the challenge (either by students or educators), and 3) focus on a real-world need. Global themes encompass significant topics such as sustainability or health. Complex problems within these themes are inconsistent, influenced by changing variables, open-ended, and include stakeholders with different values and perspectives ( Funke, 2010; Veltman *et al.*, 2019).

When students define their own specific challenge within a global theme that aims to solve part of a complex problem, ample time is needed for the divergent phase during which students explore the problem in-depth. This approach allows students to a choose a challenge aligned with their own interests, however, faculty supervision is crucial to prevent overwhelming situations due to the enormity of such an assignment. In our education, students tackled the global theme *“Healthy Urban Living*”, specifically the problem of loneliness, and were tasked to formulate an actionable challenge themselves. When faculty define the challenge, students can spend more time on problem-definition and solutions, resulting in a more action-based design of CBL. However, this approach limits students’ freedom, potentially affecting their engagement and motivation. For example, within the “ *Healthy Urban Living*” theme, a pre-defined challenge was to motivate the population to walk or cycle more. The choice between student- or faculty-defined challenges depends on the design thinking process or the client’s preference. A prerequisite of CBL, however, is that students have a certain level of creative freedom, while faculty emphasize the nature of the challenge and guide students towards developing a tangible solution within the given timeframe.

***3. Include an involved societal client***

Using a real-world problem in CBL involves addressing genuine societal needs rather than fictional scenarios created solely for educational purposes ( Crebert *et al.*, 2004; Gallagher & Savage, 2020). Involving one or multiple clients from the community who possess a complex problem requiring a solution is advantageous for CBL. This aspect closely relates to community-engaged learning (see the twelve tips by Marjadi *et al.*, 2022). Based on our experience, we emphasize the importance of managing the client relationship and establishing agreements regarding their level of involvement, including time investment and level of facilitation. We found that a higher level of client engagement facilitates smoother collaboration. At the very least, the client should be available to provide students with feedback and possess sufficient knowledge about CBL to understand the process, its objectives, and added value. Intellectual property and creative rights should also be agreed upon.

During our CBL program, students were given the opportunity to conduct an initial interview with their client, seek feedback while defining the problem, and after brainstorming sessions about potential solutions. We recommend designating one individual as the client, even if multiple people are involved, to ensure continuity. As CBL is an iterative process that emphasizes student agency, briefing the client about the varying project stages of student-teams helps manage expectations during client-student meetings. Collaboratively designing the conclusion of CBL with the client is also helpful. Possibilities include organizing an event with an external jury and award for the winning team or conducting presentations at the client’s workplace. It is crucial for students to understand if this concluding event will be included in their final assessment and how this pertains to the learning objectives of CBL.

***4. Offer a variety of educational activities***

A variety of educational activities will help to meet different goals of CBL. A not exhaustive overview of educational activities with their purpose, format options, and examples is shown in Table 1. Activities should be chosen based on learning goals, e.g., interviewing the client requires interviewing skills, therefore, a workshop that allows practice is more suitable compared to a lecture.

**Table 1.** Overview of educational activities with their purpose, format options, and examples. CBL = challenge-based learning, Q&A = questions and answers.

Educational activity	Purpose	Format options	Examples
Lectures	Provide information	Offline plenary Online content clip	Introducing the global problem
Workshops	Practice CBL skills	Offline Blended	Stakeholder interviewing skills
Inspiration sessions	Inspire students	Short presentation Intro and Q&A	Personal story about a surprising or challenging experience
Team assignments	Define challenge, problem, and solution	Document Video	Contact stakeholders Brainstorm possible solutions
Individual assignments	Stimulate personal development	Document Video	Formulating self-development goals
Coaching	Guide students through CBL and individual development	Flexible and scheduled group and individual sessions (Offline or online)	Reflecting on subjects such as teamwork and project management

Purposeful scheduling decisions enhance students’ workflow and effectivity. Examples include offering activities at consistent times, grouping them by content or type, and scheduling uninterrupted time for team and individual assignments. In our experience, students appreciated a structured approach at the beginning of the challenge, which gradually tapered off as their projects diverged, giving them increased independence and agency. Moreover, we found that using inspiration sessions to spark students’ creativity and curiosity stimulated them to proactively seek relevant context related to their challenge, rather than relying on scheduled lectures with predetermined content. This freedom and investigatory approach enhanced students’ boundary crossing motivation. Lastly, it is important to offer CBL opportunities throughout the academic year to ensure equal access for students from various disciplines. This creates a diverse student population and promotes interdisciplinary collaboration.

***5. Provide an online learning environment and versatile physical location***

CBL provides opportunities for learning activities and community building to take place online and offline. Even in completely offline education, an online learning environment is helpful for communication and information sharing. An online CBL learning environment should include: 1) a general channel for announcements and information, 2) an updatable schedule, 3) chat and video conferencing options for plenary, team, and coaching meetings, 4) educational materials (e.g., brainstorming tools, content clips, etc.) with file storage and collaborative functions, and 5) options for uploading assignments for assessment and peer-feedback.

When offline activities are scheduled, having a reserved space for the entire duration of the CBL activities provides continuity, a communal space, share of creative materials, and storing project-related items. Additionally, having a versatile location with movable and adjustable tables and chairs plus technology such as digital whiteboards or screens, facilitates the different educational activities and creative nature of CBL.

***6. Train a diverse faculty team***

Given the unpredictable nature of CBL, where students can take different directions while trying to solve complex real-world problems, we found that an involved and diverse faculty team is helpful in the design and execution of education. Faculty members should be open to embracing non-traditional ways of teaching where they do not hold all the answers but facilitate students’ search for solutions instead. Training faculty in coaching skills and enhancing their understanding of CBL fosters a community mindset and a continuous learning journey alongside the students.

The composition of the faculty team should ideally consist of members with different backgrounds and expertise, supplemented by guest faculty when specific knowledge is required. During our CBL, inspiration sessions were provided by guests from inside academia (e.g., professors), outside academia (e.g., municipality policy makers), and different disciplines (e.g., biomedical sciences and social sciences). Effective teamwork can be cultivated through reflection and feedback ( Leshed *et al.*, 2007). Therefore, we recommend organizing faculty meetings to exchange experiences and conducting feedback sessions with students to gather valuable suggestions for improvements.

***7. Emphasize interdisciplinary collaboration***

Interdisciplinary collaboration is one of the key features of CBL ( Gallagher & Savage, 2020) and necessary for solving complex (health) problems ( Bridges *et al.*, 2011). Team formation can play an important role in this process and can be approached in two ways. Students can be given the opportunity to form their own teams, or teams can be formed by faculty. It is recommended that teams consist of a minimum of four members, although the number can be adjusted based on the timeframe and complexity of the challenge, and teams can consist of students from different academic levels (e.g., undergraduates, graduates, and lifelong learners). Given the demanding nature of CBL, teams should aim to be diverse (e.g., mixing educational backgrounds, personality traits, generic skills, and preferred team roles). This will ensure a variety of perspectives and expertise and give students the opportunity to practice interdisciplinary collaboration in preparation for post-graduation work environments.

The interdependency among team members during CBL is high. Therefore, sufficient attention and time should be dedicated to team building. Incorporating team building activities, such as competing with other teams in short challenges where communication is key or getting to know each other games, helps with creating trust between team members ( Hastings *et al.*, 2018).

***8. Create a community***

In addition to interdisciplinary collaboration within student-teams, it is beneficial to foster a sense of community under all participants in the CBL process, including faculty and coaches. Building a community provides students with a supportive environment and a safe space for reflection, knowledge sharing, learning, and collective growth. Both formal and informal meetings, scheduled during and outside of educational hours, can contribute to community building. Ramani *et al.* (2021) outlined three phases for developing a community of practice: 1) establish, 2) grow, and 3) sustain. Including communal activities such as joint breaks, lunch walks, and energizers in the schedule and encouraging faculty involvement can foster a sense of togetherness. Moreover, students can be encouraged to contribute to community building by suggesting and organizing activities, such as seminars, sports tournaments, or other social gatherings.

Faculty members can enhance community building by being visible and available for students, e.g., by being present in the physical learning space with the students while they work. Beginning and ending each day together also offers opportunities for faculty to reiterate learning objectives and check in with students to ensure they have everything they need to move forward with their projects.

***9. Help students deal with uncertainty***

Students engaging in CBL often encounter the unfamiliarity of its open character, where the challenge and its solution are not predetermined, and faculty do not possess all the answers ( Nichols *et al.*, 2016). It is important for students to learn how to navigate this uncertainty, and faculty can support them by stimulating control behaviors, task monitoring, and self-monitoring ( Osman, 2010).

Control behaviors involve actions aimed at achieving a goal, which in CBL refers to problem-solving. Faculty can assist students by providing them with tools and instruction for the CBL process, such as discussing the CBL or design thinking structure, offering skill workshops, and sharing examples from previous student-teams. Task monitoring entails understanding the task, so faculty must provide clear instructions and expectations to students. Acknowledging that uncertainty and making mistakes are part of the innovative and creative character of CBL ( Gallagher & Savage, 2020) can also be addressed in instruction, with faculty ensuring that this topic remains open for discussion within the community. Self-monitoring involves assessing task progress and the effectiveness of actions. One way to do this is through reflection. Additionally, feedback from others is a useful tool ( Leshed *et al.*, 2007). Lastly, faculty should clearly communicate how and to what extent students will be assessed.

***10. Coach students and provide feedback on their development***

Supporting students requires recognizing their diverse backgrounds, experiences, knowledge, and (generic) skills, which makes a one-size-fits-all approach challenging. Coaching plays a vital role in CBL as it allows for individualized support tailored to each student’s needs. A coach can provide guidance to students both individually and within teams, asking critical questions and facilitating opportunities for them to work towards their goals and explore different team roles, preparing them for post-graduation. A coach can help students navigate uncertainty by helping them find structure within the fluidity of CBL. Feedback is highlighted as a valuable learning mechanism in CBL ( Gómez *et al.*, 2022), and a coach can provide direct feedback on students’ actions or engage in reflective discussions with students regarding feedback from others.

***11. Stimulate reflection***

Reflection, as defined by Sandars (2009), is a process that enhances understanding of a specific situation and oneself to inform future behavior. It has been linked to an increased ability to deal with complex problems ( Ramaley, 2014; Rogers, 2001). To introduce reflection to our students, we conducted a workshop where we discussed its importance for personal and professional growth ( Sandars, 2009), and shared the experiential learning cycle, consisting of four phases: 1) describing the experience, 2) reviewing the experience, 3) learning from the experience, and 4) experimenting in a new experience ( Kolb, 1984).

Throughout the educational process, students were provided with prompts to start the reflective process ( Aronson, 2011), such as reflection topics (e.g., trust, resilience, passion) and scheduled disengagement moments (e.g., guided meditation, physical exercises, walking in silence). Linking these prompts to specific educational activities, such as trust and team brainstorming, helps the integration of reflection and education. Reflection can also be stimulated by others ( Sandars, 2009), with coaches playing a role in helping students select relevant situations for reflection, analyzing those situations, and formulating alternative behaviors. We incorporated both short reflections throughout the CBL process and a more comprehensive reflection exercise at the end, allowing students to process their learning. As research indicates no advantage of one format over the other ( Aronson, 2011), students were free to choose their own format for their final reflection, such as a written document or video. In addition to individual reflection, we also fostered reflection as a community by organizing general feedback moments.

***12. Design appropriate assessment***

In CBL, it is important to employ appropriate assessment methods for both team and individual development ( Kohn Rådberg *et al.*, 2020). Team development is often assessed through presentations of end results to the community ( Gallagher & Savage, 2020), however, each team’s challenge specifics such as problem-definition and proposed solutions can be assessed as well. Individual development includes personal learning goals set throughout the educational process ( Gallagher & Savage, 2020).

Tackling complex problems with societal impact requires students to apply their domain-specific knowledge and skills flexibly while employing generic skills in different contexts. CBL, with its diverse challenges, stakeholders, and educational activities, provides an ideal platform for developing professional skills such as collaboration, boundary crossing, and communication. These skills can be assessed in detail. For instance, we used personal development plans in which students reflected on and described their skills development ( Baker *et al.*, 2014). A personal development plan can include students’ personal learning goals, structured reflection reports, peer-feedback, and teacher observations. Students are assessed on the depth and clarity of their description and their growth regarding professional skills. For more assessment suggestions, see the twelve tips by Van Der Vleuten, Schuwirth, Driessen, Govaerts, and Heeneman (2015) and the CBL framework by Nichols, Cator, & Torres (2016).

## Conclusion

Being confronted with complex real-world challenges is part of being a health professional. Developing and practicing complex problem-solving should therefore be part of biomedical education, and CBL is a pedagogy that can support this process. The twelve tips described in this paper are inspired by multiple CBL educational activities within our Graduate School of Life Sciences but can be adapted to other biomedical programs, as design considerations are described in a broader context, and practical examples are provided. By integrating CBL in biomedical education, students are challenged to develop their complex problem-solving skills and are given the opportunity to involve multiple stakeholders from inside and outside academia, such as patients and industry, to become the health professionals of the future.

## Data Availability

No data are associated with this article.
