# Supplementary material for: Practical tips for organizing challenge-based learning in biomedical education
Source: MedEdPublish (2016). 2023 Nov 8;13:271. [Version 1] doi: 10.12688/mep.19755.1 (PMC10739181; doi:10.12688/mep.19755.1)
Supplement: Peer review history

# Reviewer report: Reviewer response for version 1

**DOI:** 10.21956/mep.21169.r35463

**Published:** 2023-12-21

**Authors:** Rita Dadiz

**Affiliations:** 1 University of Rochester Medical Center, Rochester, USA

The authors present tips for educators who are helping learners utilize challenge-based learning (CBL) as an approach to design strategies that address real-world problems.  They base their tips on several years of experience using CBL in biomedical education, which is particularly helpful whenever they provide examples.  I have a few comments and clarifying questions as follows:

 Tip 1: It may be helpful for those who are new to CBL to understand how the different CBL frameworks are similar and how they may differ. Are there components that these frameworks all share? Would specific frameworks be better suited for particular types of problems or teams? Perhaps providing a concise table would be illustrative. 

 Tips 2 and 10: I appreciate that the authors provided healthy urban living as an example of a global theme. In these sections, they discuss ways for educators to provide more or less support, depending on what their learners need. In a large group setting where there may be multiple teams working simultaneously, is there usually an assigned faculty coach or mentor for each team who would gauge progress?

 Tip 6: There may also be an opportunity for faculty who are new to CBL to be paired with those who are more experienced to allow for experiential faculty development.

If evidence from practice is presented, are all the underlying source data available to ensure full reproducibility?

Not applicable

Is the topic of the practical tips discussed accurately in the context of the current literature

Yes

Are all factual statements correct and adequately supported by citations?

Yes

Are the conclusions drawn balanced and justified on the basis of the presented arguments?

Yes

Are arguments sufficiently supported by evidence from the published literature and/or the authors’ practice?

Yes

Reviewer Expertise:

Medical education

I confirm that I have read this submission and believe that I have an appropriate level of expertise to confirm that it is of an acceptable scientific standard.

# Reviewer report: Reviewer response for version 1

**DOI:** 10.21956/mep.21169.r35272

**Published:** 2023-11-30

**Authors:** Jasmina Lazendic-Galloway

**Affiliations:** 1 Eindhoven University of Technology, Eindhoven, The Netherlands

The paper presents practical tips for educators who might be considering the implementation of CBL. The authors draw guidelines on how to organise a CBL course based on their extensive practical experience with CBL. The paper offers a succinct overview of the key elements of practice that are important for a CBL course, with useful ideas, tools and examples. Where applicable, the authors draw parallels with or recommendations from the existing literature.

 I have a few minor suggestions for improving/clarifying the text, which are given below:

 Abstract

 1) Our aim is to promote CBL in biomedical education and **educators with the adoption and implementation process in order** to help students acquire…

 Introduction, second paragraph

 2) In their international literature review, Gallagher and Savage (2020) provide a summary of the commonly **identifie**d **[instead of "agreed upon"] **characteristics,…

 Twelve tips - 2. Select a complex real-world problem

 3) Global themes encompass significant topics such as sustainability or health. Complex problems within these themes are **ill-defined [instead of "inconsistent"]**,…

 4) A prerequisite of CBL, however, is that students have a certain level of creative freedom, while **the** **faculty’s role is to emphasize [instead of "faculty"]** the nature of the challenge and guide students towards developing a tangible solution within the given timeframe.

If evidence from practice is presented, are all the underlying source data available to ensure full reproducibility?

Not applicable

Is the topic of the practical tips discussed accurately in the context of the current literature

Yes

Are all factual statements correct and adequately supported by citations?

Yes

Are the conclusions drawn balanced and justified on the basis of the presented arguments?

Yes

Are arguments sufficiently supported by evidence from the published literature and/or the authors’ practice?

Yes

Reviewer Expertise:

STEM education, challenge-based learning, adoption of innovative practices

I confirm that I have read this submission and believe that I have an appropriate level of expertise to confirm that it is of an acceptable scientific standard.
